# Interacting with Obstacles Using a Bio-Inspired, Flexible, Underactuated Multilink Manipulator

**DOI:** 10.3390/biomimetics9020086

**Published:** 2024-02-01

**Authors:** Amit Prigozin, Amir Degani

**Affiliations:** 1 Faculty of Civil and Environmental Engineering, Technion-Israel Institute of Technology, Haifa 3200003, Israel; 2 Technion Autonomous Systems Program (TASP), Technion-Israel Institute of Technology, Haifa 3200003, Israel

**Keywords:** robotics, bioinspired and biomimetic robotics, soft robots, robot manipulation

## Abstract

With the increasing demand for robotic manipulators to operate in complex environments, it is important to develop designs that work in obstacle-rich environments and can navigate around obstacles. This paper aims to demonstrate the capabilities of a bio-inspired, underactuated multilink manipulator in environments with fixed and/or movable obstacles. To simplify the system design, a single rotational actuator is used at the base of the manipulator. We present a modeling method for flexible, multilink underactuated manipulators, including their interaction with obstacles. We also demonstrate how to plan a trajectory for the manipulator in environments with fixed obstacles. The robustness of the manipulator is examined by analyzing the effects of uncertainty in its initial state and the position of obstacles. Next, we demonstrate the performance of the manipulator in environments with movable obstacles and show the advantages of controlling the obstacles’ radii and positions. Lastly, we showcase the process of picking up an object in workspaces with obstacles. All the findings are supported by simulations as well as hardware experiments.

## 1. Introduction

Robotic manipulators have become indispensable in various industries ranging from car assembly to fruit harvesting in orchards. However, these manipulators often must operate in complex environments, where multiple obstacles surround them. These obstacles impose constraints on the arm’s trajectory, reducing the accessible workspace and making end-effector trajectory planning significantly more complicated.

To navigate around obstacles in the workspace, it is often necessary to increase the number of active degrees of freedom (DOFs) of the manipulator. By increasing the number of DOFs, the manipulator gains the ability to bypass obstacles and reach its desired target pose. However, increasing the number of active actuated joints also increases the complexity of the manipulator design, its cost, and the complexity of trajectory planning.

The elephant trunk is a sophisticated organ that can perform complex tasks while maintaining a minimal design. By using only twelve basic muscles, the trunk is capable of performing most of its tasks, from carrying heavy loads to performing gentle object manipulations [1]. Over the years, studies have shown that evolution has indeed optimized the number of active degrees of freedom in the elephant trunk [2]. During the development of the current manipulator, the distinctive features of the elephant trunk served as a source of inspiration, guiding us toward a more optimized solution suitable for a wide range of tasks.

This work proposes a novel approach for employing manipulators in environments with obstacles using a multilink, bio-inspired underactuated manipulator. To simplify the control of the system, we chose to use a single, active, actuated revolute joint at the base of the manipulator. As torque is applied to the first link of the manipulator, the entire manipulator deforms due to the internal forces in the joints.

Traditional trajectory-planning algorithms for industrial manipulators focus on avoiding obstacles to prevent damage to the manipulator or its environment. However, when using flexible manipulators, interactions with the environment can occur without risking damage to either. Furthermore, such interactions might in fact help reduce the uncertainty in the end-effector’s position, leading to an improved accuracy and robustness of the manipulator. In addition, by controlling the positions and radii of the obstacles, we can increase the variety of tasks that the manipulator can perform. These benefits will be discussed in greater detail in this manuscript.

Planning trajectories for a manipulator with many links and a complex motion is challenging without a predetermined analytic input torque command to reach a desired configuration. In our work, we adopted a stochastic optimization scheme to find the torque command necessary to reach a desired target point. This approach allows us to overcome the complexity associated with trajectory planning for a high-degree-of-freedom manipulator, and effectively control its motion to achieve the desired configuration.

**Figure 1:**
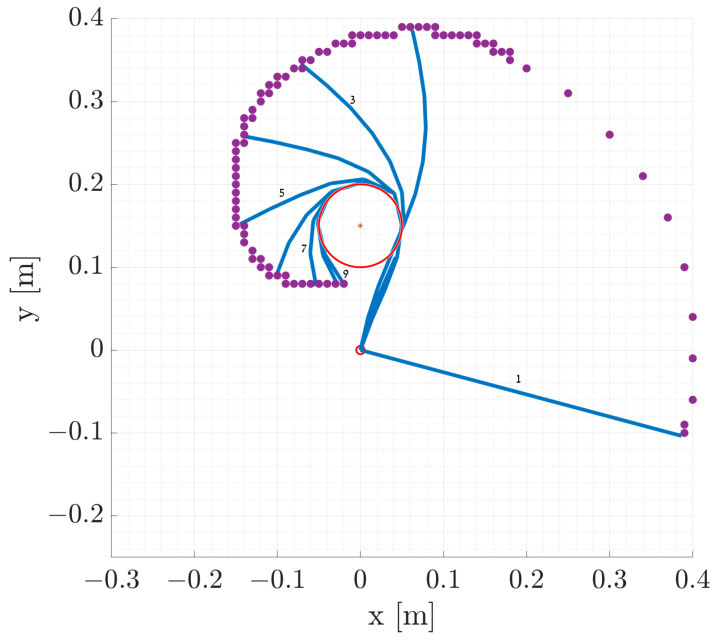
A simple simulation of a multilink, underactuated manipulator (shown in blue lines) interacting with an obstacle (shown in red circle). The manipulator sequence over time is denoted by the numbers at the end of the manipulator, while the trajectory of the end-effector is represented by purple dots. The simulation duration is 0.5 s.

In Figure 1, we present a simple simulation of a multilink manipulator’s end-effector trajectory as it interacts with an obstacle. In this simulation, a constant torque is applied to the base link, and the trajectory of the end-effector is recorded. This figure effectively demonstrates the model’s ability to simulate the manipulator’s interaction with its environment, showcasing how the manipulator’s high number of links enables it to reach the opposite side of the obstacle.

This work builds upon our previous conference publication, where we studied the dynamic manipulation of deformable manipulators, albeit in obstacle-free environments, and developed a method for trajectory planning to reach desired targets [3].

In this paper, we present a comprehensive study of the system’s behavior by developing a dynamic model. The effectiveness of this model will be assessed through a series of simulations in environments with fixed obstacles that will be subsequently validated through experiments.

In this work, we evaluate the robustness of the system against uncertainty in the manipulator’s initial angle and input torque. Later, we will evaluate the performance of the manipulation in environments with movable obstacles. We will also demonstrate a straightforward pickup process in both environments with fixed and movable obstacles. Our primary objective is to highlight how a simple, flexible, underactuated manipulator can perform just as effectively as a rigid manipulator in terms of its workspace. In addition to this, we will also examine the system’s sensitivity to variations in the manipulator’s initial angle, input torque, and obstacle position. We aim to provide a detailed analysis that demonstrates how a simple manipulator can offer a comparable performance while remaining adaptable to various environmental conditions.

## 2. Related Work

As mentioned, this paper describes a bio-inspired, underactuated multilink manipulator. Drawing inspiration from the elephant trunk in our design, we initiate our exploration by delving into various trunk models, such as the kinematic continuum model presented by Wilson [4] and the dynamic model articulated by Mishra [5]. Over the years, numerous robotic systems have been developed, all inspired by elephant trunks, ranging from shape memory, alloy-based systems [6] to more complex mechanisms like the one presented by Huang [7], and even different types of unique grippers [8]. These works collectively underscore the inherent advantages of the elephant trunk and demonstrate how a manipulator inspired by such principles can reap similar design benefits.

To provide context, we begin by reviewing the literature on classic serial rigid robots. A vast amount of research has been devoted to rigid robotic arm manipulation, with early work mostly focusing on manipulation in simple, obstacle-free environments. For example, in 1983, Lin et al. published a paper on trajectory optimization in the joint space [9], while in 1988, Kyriakopoulos et al. presented a paper on trajectory planning while maintaining minimum jerk [10]. As the interest in manipulation in environments with obstacles grew, researchers such as Zavlangas and Tzafestas explored the navigation around obstacles using fuzzy logic in 2000 [11], and in 2016, Rubio proposed an efficient optimal trajectory generation without obstacle collisions [12]. These works contributed significantly to the development of rigid robot manipulation, but rigid manipulators have inherent limitations that have driven research interest towards other solutions such as deformable manipulators.

It is essential to have an accurate model of the manipulator as a first step. Since this work focuses on multilink manipulators, it is only natural to describe the system as a set of ordinary differential equations (ODEs), where each equation represents the dynamic model of one link. Several researchers have proposed methods for modeling deformable manipulators using ODEs. Suzuki et al. formulated a manipulator model as a set of ODEs in their work on casting and winding a tethered projectile around an obstacle [13]. Yamakawa et al. used a serial manipulator to perform various tasks on a deformable manipulator, such as folding a piece of fabric, tying a knot on a rope, and manipulating a gymnastic ribbon [14,15,16]. Mochiyama cast an end-effector connected by a flexible elastic rope to the ground using a pneumatic system, and aimed it at a desired target location [17]. Fagiolini developed a simple two-DOF system that spins an end-effector at a high velocity, and then releases and casts it to a target [18]. In all the above-mentioned works, the authors model the system using ODEs by describing the deformable object as a set of links. In our work, we chose a similar approach.

While the previous works focused on manipulating deformable objects as multilink manipulators, none of them addressed the interaction between the manipulator and obstacles in the environment. In contrast, Hatton et al. used a simple manipulator to cast a projectile attached to a rope and wrap it around a horizontal pole [19], and Yamakawa demonstrated the high-speed manipulation of a whip by wrapping it around a pole while using camera feedback [20]. However, in these examples, obstacles were only used as target points and did not affect the trajectory of the manipulator. On the other hand, we came across work like that of Liu et al., in which an inflatable flexible manipulator is controlled to navigate inside large spaces quasi-statically [21,22]. Another similar quasi-static manipulator is the pneumatic bending actuator of Wolf et al. [23]. In our work, we address the manipulation of dynamic and underactuated manipulators in environments with obstacles, an area that has not been explored in previous research on deformable manipulators.

Given the interaction of the manipulator with the obstacles, it is necessary to characterize the nature of the contact between them. Contact modeling has been the subject of numerous studies over the years. The Hertz model, which dates back to the 19th century, assumes a small contact area with a single asperity at the contact point [24]. In this model, the contact bodies are assumed to be elastic, with a linear behavior and without friction, resulting in the presence of only normal forces. Goodman expanded on the model and studied the effect of friction without tangential loads in the Hertz model [25]. Subsequently, Mossakovski and Spence conducted separate studies on the complete solution, including shear tractions [26,27,28]. Greenwood and Williamson also presented a study on the elastic contact of uncoupled multi-asperity [29].

To streamline the model and reduce the computation time in our work, we make the simplifying assumption of pure plastic contact between the manipulator and the obstacles. Upon initial contact with an obstacle, a virtual spring-damper mechanism is established at the contact point to impose constraints on the system, while the remaining portion of the manipulator remains free to move. We will subsequently verify this assumption through our experiments.

## 3. Methodology

To start, we establish the model and governing equations that describe the behavior of the system utilizing the ODE approach, similar to our preliminary work [3]. We model the deformable manipulator as a set of short, rigid links with a constant linear viscous dissipation between them. The model is based on several assumptions: (1) the system is planar, normal to gravity, and therefore, gravity does not impact the system; (2) the length of each link, L, is the same for all links and is significantly longer than its other two dimensions; (3) the links are nonelastic, i.e., the manipulator maintains a constant length; (4) the manipulator has a constant cross-sectional area and density; and (5) the mass of each link, m, is concentrated at its geometric center.

To formulate the dynamic equations, we adopt the Lagrangian approach. We start by defining the position vector of each link:

(1) $$\left(\begin{array}{c} P_{x_{i}}\\ P_{y_{i}} \end{array}\right)=\left(\begin{array}{c} x_{i-1}+L\cdot sin\left(\theta_{i}\right)\\ y_{i-1}-L\cdot cos\left(\theta_{i}\right) \end{array}\right)$$

where, L is the link length, $\theta_{i}$ is the link’s angle with respect to the *x*-axis of the world frame, and $x_{i-1}$ and $y_{i-1}$ are the position coordinates of the distal end of the previous link. The reference point for the manipulator is the base of the first link, which will be referred to as (0,0) for convenience. To calculate the velocity of each link and find the kinetic energy of the system, we take the derivative of the position vector over time:

(2) $$T=\sum \left(\frac{1}{2}mv_{i}^{2}\right).$$

In this model, we assume that each link can be approximated as a point mass; therefore, the links do not have a tensor of inertia. The symbol m represents the mass of each link, and *v* represents the magnitude of the velocity vector of each link.

As the manipulator operates in a two-dimensional environment, we do not need to formulate the potential energy expression. The expression for the energy dissipation between the N links is

(3) $$D=\frac{1}{2}b\left({\dot{\theta }}_{1}^{2}+\sum_{i=2}^{N}{\left({\dot{\theta }}_{i}-{\dot{\theta }}_{i-1}\right)}^{2}\right),$$

where *b* is the dissipation coefficient, which we set to be constant for all links.

We now derive the equations of motion using the Lagrangian formulation as

(4) $$\frac{d}{dt}\left(\frac{\partial T}{\partial \dot{q}}\right)-\frac{\partial T}{\partial q}+\frac{dD}{d\dot{q}}=\tau_{ext}.$$

Equation (4) incorporates the external moment vector $\tau_{ext}$ and the manipulator state variable, q, which includes the angle and angular velocity of each link, where $q={\left[\theta_{1}\ldots \theta_{N},{\dot{\theta }}_{1}\ldots {\dot{\theta }}_{N}\right]}^{T}$.

By combining Equations (2) and (3) into Equation (4), we arrive at Equation (5), which presents the general equation structure for *N* first order ODEs in matrix form:

(5) $$M\left(q\right)\ddot{q}+B\dot{q}+C\left(q\right)\cdot \dot{q}\cdot \dot{q}=\tau ,$$

where, *M* is the inertia matrix:

$$M=\left[\begin{array}{cccccc} -NmL^{2} & -\left(N-1\right)mL^{2}cos\left(\theta_{1}-\theta_{2}\right) & -\left(N-2\right)mL^{2}cos\left(\theta_{1}-\theta_{3}\right) & \ldots & -2mL^{2}cos\left(\theta_{1}-\theta_{N-1}\right) & -mL^{2}cos\left(\theta_{1}-\theta_{N}\right)\\ -\left(N-1\right)mL^{2}cos\left(\theta_{2}-\theta_{1}\right) & -\left(N-1\right)mL^{2} & -\left(N-2\right)mL^{2}cos\left(\theta_{2}-\theta_{3}\right) & -\left(N-3\right)mL^{2}cos\left(\theta_{2}-\theta_{4}\right) & \ldots & -mL^{2}cos\left(\theta_{2}-\theta_{N}\right)\\ -\left(N-2\right)mL^{2}cos\left(\theta_{3}-\theta_{1}\right) & -\left(N-2\right)mL^{2}cos\left(\theta_{3}-\theta_{2}\right) & -\left(N-2\right)mL^{2} & -\left(N-3\right)mL^{2}cos\left(\theta_{3}-\theta_{4}\right) & \ldots & -mL^{2}cos\left(\theta_{3}-\theta_{N}\right)\\ \vdots & \ddots & \ddots & \ddots & \ddots & \vdots \\ -2mL^{2}cos\left(\theta_{N-1}-\theta_{1}\right) & -2mL^{2}cos\left(\theta_{N-1}-\theta_{2}\right) & \ldots & -2mL^{2}cos\left(\theta_{N-1}-\theta_{N-2}\right) & -2mL^{2} & -mL^{2}cos\left(\theta_{N-1}-\theta_{N}\right)\\ -2mL^{2}cos\left(\theta_{N}-\theta_{1}\right) & -2mL^{2}cos\left(\theta_{N}-\theta_{2}\right) & -2mL^{2}cos\left(\theta_{N}-\theta_{3}\right) & \ldots & -2mL^{2}cos\left(\theta_{N}-\theta_{N-1}\right) & -2mL^{2} \end{array}\right],$$

B is the dissipation, tridiagonal matrix:

$$B=\left[\begin{array}{cccccc} 2b & -b & 0 & 0 & \ldots & 0\\ -b & 2b & -b & 0 & \ldots & 0\\ 0 & -b & 2b & -b & \ldots & \vdots \\ \vdots & \ddots & \ddots & \ddots & \ddots & 0\\ 0 & \ldots & 0 & -b & 2b & -b\\ 0 & \ldots & 0 & 0 & -2b & 2b \end{array}\right],$$

C includes all the nonlinear components:

$$C=\left[\begin{array}{cccccc} 0 & \left(N-1\right)mL^{2}sin\left(\theta_{1}-\theta_{2}\right) & \left(N-2\right)mL^{2}sin\left(\theta_{1}-\theta_{3}\right) & \ldots & 2mL^{2}sin\left(\theta_{1}-\theta_{N-1}\right) & mL^{2}sin\left(\theta_{1}-\theta_{N}\right)\\ \left(N-1\right)mL^{2}sin\left(\theta_{2}-\theta_{1}\right) & 0 & \left(N-2\right)mL^{2}sin\left(\theta_{2}-\theta_{3}\right) & \left(N-3\right)mL^{2}sin\left(\theta_{2}-\theta_{4}\right) & \ldots & mL^{2}sin\left(\theta_{2}-\theta_{N}\right)\\ \left(N-2\right)mL^{2}sin\left(\theta_{3}-\theta_{1}\right) & \left(N-2\right)mL^{2}sin\left(\theta_{3}-\theta_{2}\right) & 0 & \left(N-3\right)mL^{2}sin\left(\theta_{3}-\theta_{4}\right) & \ldots & mL^{2}sin\left(\theta_{3}-\theta_{N}\right)\\ \vdots & \ddots & \ddots & \ddots & \ddots & \vdots \\ 2mL^{2}sin\left(\theta_{N-1}-\theta_{1}\right) & 2mL^{2}sin\left(\theta_{N-1}-\theta_{2}\right) & \ldots & 2mL^{2}sin\left(\theta_{N-1}-\theta_{N-2}\right) & 0 & mL^{2}sin\left(\theta_{N-1}-\theta_{N}\right)\\ 2mL^{2}sin\left(\theta_{N}-\theta_{1}\right) & 2mL^{2}sin\left(\theta_{N}-\theta_{2}\right) & 2mL^{2}sin\left(\theta_{N}-\theta_{3}\right) & \ldots & 2mL^{2}sin\left(\theta_{N}-\theta_{N-1}\right) & 0 \end{array}\right],$$

and τ is the external torque vector:

$$\tau =\left[\begin{array}{c} \tau_{ext}\\ 0\\ 0\\ 0\\ \vdots \\ 0 \end{array}\right].$$

In Section 4 and Section 6, we demonstrate the accuracy and representativeness of this model through a series of simulations and experiments.

Additionally, to develop the mathematical model for the multilink manipulator, we also created a contact model for the interaction between the manipulator and obstacles. When the manipulator comes into contact or penetrates an obstacle, we establish a spring-damper mechanism between the center of the obstacle and the contact point on the manipulator, which applies an external force on the manipulator. To create a stiff constraint and minimize the relative movement at the contact point, we chose a spring constant of $50\times 10^{5}\;\left[N/m\right]$ and a damping constant of $50\times 10^{3}\;\left[N\cdot s/m\right]$.

Since the ODE input is an external torque on the joints, the external forces from the obstacles need to be mapped. This conversion requires the use of a virtual manipulator with fewer links, whose end-effector is positioned at the contact point. By calculating the Jacobian matrix of the virtual manipulator, we can use Equation (6) to translate the external forces acting on the virtual end-effector into torques on each joint between the contact point and the manipulator’s base as

(6) $$\bar{\tau }=\left[\begin{array}{c} \tau_{1}\\ \tau_{2}\\ \vdots \\ \tau_{N} \end{array}\right]={J_{i}\left(\bar{q}\right)}_{n\times 6}^{T}\cdot \left[\begin{array}{c} \bar{F}\\ \bar{T} \end{array}\right].$$

## 4. Simulations

### 4.1. Fixed Obstacles

Once we established the mathematical model of the system, we could proceed to evaluate it in a series of simulations. As previously noted, the primary objective of this paper is to showcase a control technique for a bio-inspired, multilink manipulator, as well as to observe how it interacts with obstacles. All the initial simulations were performed with fixed obstacles.

To verify the mathematical model, we performed our first simulation. Figure 1 provides an example of a general simulation with a fixed obstacle interaction and shows several snapshots of the manipulation motion. The purple dots in the figure represent the trajectory of the end-effector. For this simulation, a deformable object measuring 40 cm in length and divided into 10 links was used, with an input torque of 0.085 Nm. The manipulator’s initial angle was set at −15° with respect to the world frame *x*-axis, and its angular velocities and accelerations were both zero.

As shown in Figure 1, the manipulator trajectory is as expected. This simulation is used to verify that the framework is indeed working as intended, including its interaction with the environment, and we can now proceed to more complex simulations.

The next phase of our assessment involved testing the sensitivity of the system. The aim of this test is to verify our hypothesis on whether interacting with fixed obstacles can reduce the uncertainty in the manipulator end-effector position. We tested two types of uncertainty in the system: uncertainty in the **initial conditions** (i.e., initial angle and torque command) and uncertainty in the **obstacle position**. The purpose of this phase was to evaluate the degree to which these parameters impact the manipulator’s ability to reach its target.

To accomplish this, we created a simple environment consisting of a single obstacle and examined the effects of deviations in the aforementioned parameters on the end-effector’s trajectory. To begin, we simulated errors in both the input torque and the initial angle of the manipulator simultaneously. We conducted 10 simulations with random noise of up to 50% from the nominal values of 0.028 Nm and −15 degrees.

**Figure 2:**
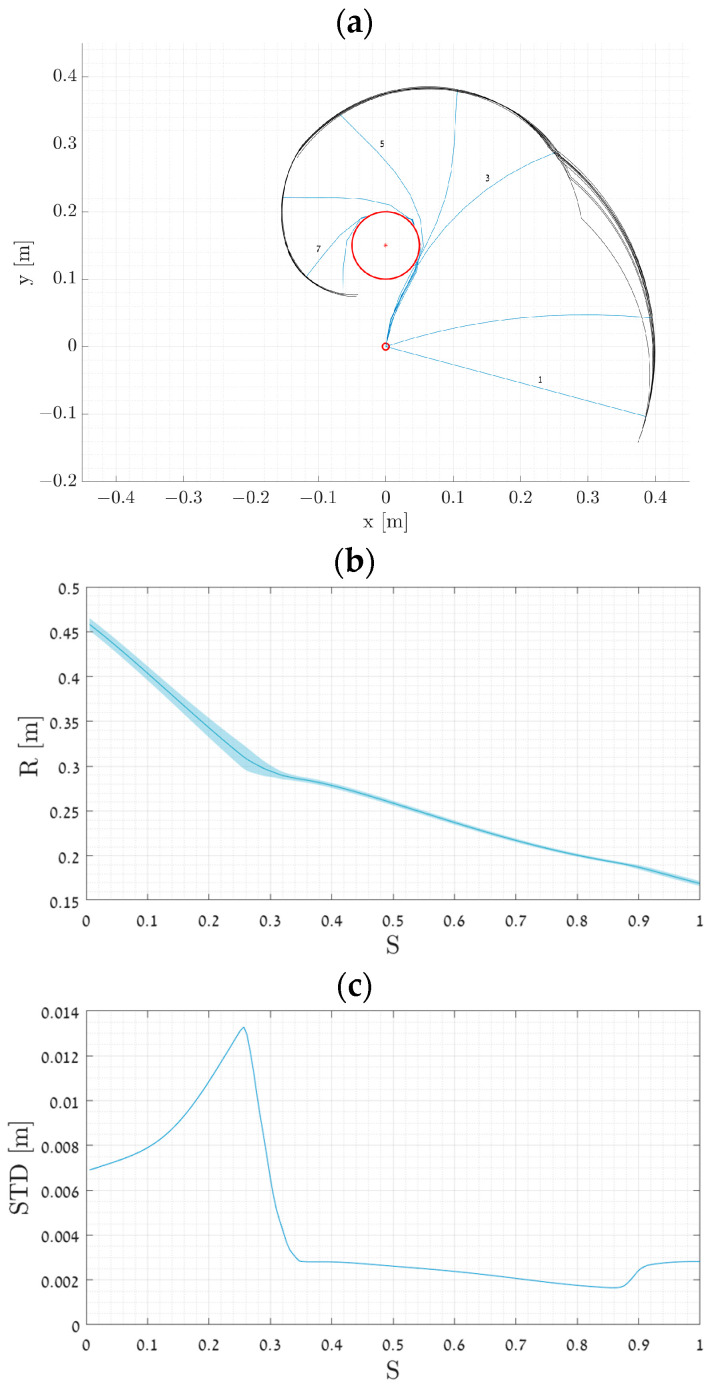
Effect of initial condition uncertainty on the system’s robustness during obstacle interaction. (**a**) Trajectories in Cartesian plane under initial condition uncertainty (i.e., initial torque and angle). Black lines represent end-effector trajectory, red circle represents obstacle, and blue lines represent manipulator snapshots. Uncertainty in the system decreases after the interaction with the obstacle. (**b**) Same data in polar coordinates representation: radial position of the end-effector along S with light blue SD envelope. (**c**) Standard deviation along S, with reduced uncertainty after obstacle interaction.

Figure 2a shows the trajectories in the Cartesian plane. As illustrated in the figure, the trajectories diverge at the start of the motion due to variations in the initial conditions. However, as the manipulator interacts with the obstacle, the uncertainty in the system decreases, and the trajectories converge into a single trajectory.

Figure 2b presents a distribution of all trajectories. The *x*-axis denotes the parametrization of the trajectory’s normalized length (denoted by S), where S = 0 corresponds to the start of the trajectory, and S = 1 corresponds to its end. The *y*-axis represents the radial distance between the manipulator’s origin and the end-effector. The brighter outline in the figure represents the standard deviation (SD) at each S, while the solid line depicts the mean value.

In Figure 2c, we can see the SD along with the trajectory parametrization S. As shown in the figure, the SD becomes smaller over time as the trajectories converge. We can observe the impact moment of the manipulator with the obstacles around S=0.3, as the variance decreases sharply due to the obstacle.

The simulation was conducted multiple times with varying noise amplitudes. The results are summarized in Table 1. For each noise amplitude, the table displays the maximum SD along the S parameter and the difference between the SD before and after the interaction with the obstacle.

**Figure 3:**
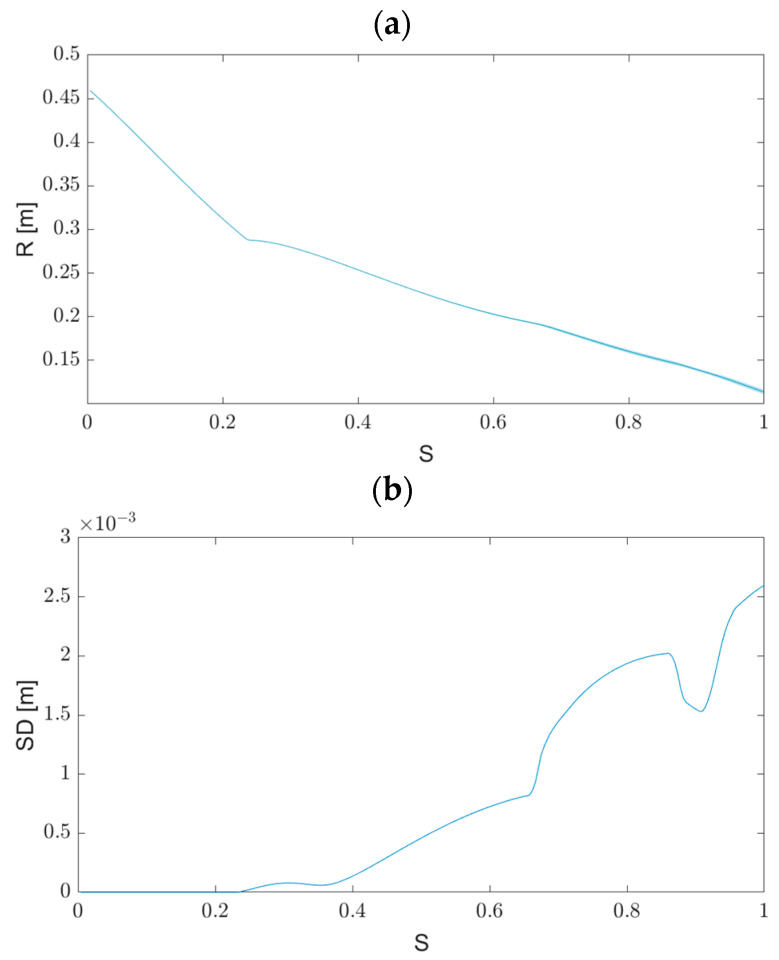
(**a**) The end-effector trajectories in Cartesian coordinates under uncertainty in the obstacle position. It can be observed that the uncertainty in the system increases after the manipulator interacts with the obstacle. (**b**) The standard deviation of the end-effector position along S under uncertainty in the obstacle location. It is shown that the uncertainty in the position of the end-effector increases after the interaction with the obstacle.

Moving on, we proceeded to simulate uncertainty in the obstacle location. In these simulations, the initial conditions were precise, without any deviations. The simulation setup and parameters were similar to the previous one. Figure 3a shows the various end-effector trajectories when the absolute obstacle location is subject to a 1% error. The red dashed line depicts the possible boundary of the obstacle. These simulations demonstrate that even a minor deviation of 1% in the obstacle location can cause the trajectories to diverge significantly. Figure 3b displays the SD of the different trajectories along S. We observe that at S = 0.4, the object interacts with the obstacle, leading to an increase in the SD.

To conclude, we found that different error sources have varying impacts on the system’s stability. While the system is robust to initial condition variances of up to 50%, it is highly sensitive to deviations in the obstacle location.

Up to this point, we have focused on examining the dynamic model and the impacts of deviations in the initial conditions. However, due to the system’s chaotic characteristics, planning a trajectory to reach a predetermined target point is challenging. To address this, we will present a method for determining the optimal initial angle and input torque required to achieve a specific point in the workspace.

**Figure 4:**
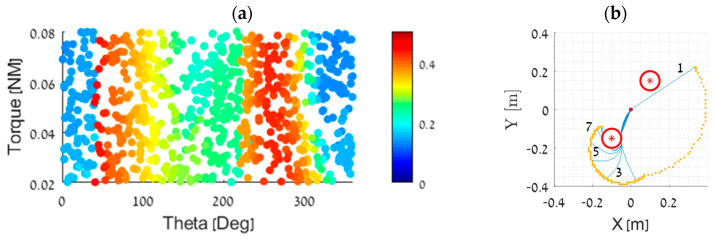
(**a**) The end-effector minimal distance from target mapping as a function of different initial conditions. The color bar on the right shows the values of the minimum distance (in meters) along the trajectory for each combination of initial angle and torque command. (**b**) Snapshots of the end-effector trajectory are shown with the initial angle and torque command obtained from the genetic algorithm. The end-effector accurately passes through the target point at (−0.15, −0.1). As above, red circle represents the obstacle, and blue lines represent the manipulator snapshots.

First, we selected a simple environment with two fixed obstacles, as shown in Figure 4b, and arbitrarily chose a target point at$\;\left(-0.15,-0.1\right)$. Figure 4a illustrates the mapping between the input parameters (initial angle and torque command) and the minimum Euclidian distance between the end-effector and the target point along the trajectory. The red dots indicate that the end-effector did not come close to the target point, while the blue dots indicate that the input parameters resulted in the end-effector being in close proximity to the target point along the trajectory.

We utilized a stochastic optimization method, the genetic algorithm [30], to determine the optimal initial conditions for the manipulator to bring the end-effector as close as possible to the target along its trajectory. To focus on dynamic solutions, we limited the algorithm to torques of $\left|\tau \right|>0.02\;\mathrm{Nm}$. We set the population size to 50 individuals and the maximum number of generations to 500, with a limit of 50 stall generations (i.e., 50 consecutive generations without a significant improvement). The system successfully converged within 150 iterations to a solution $\left(\tau ,\theta \right)=\left(0.0319,\;0.5827\right)$ (θ measured in radians), with a minimal distance of approximately 0.0074 [m]. As illustrated in Figure 4a, this solution is located around the global minimum. In Figure 4b, we show a simulation using the initial condition configuration extracted from the genetic algorithm. It is evident that the manipulator utilizes the obstacle as a pivot point to achieve great accuracy in reaching the target point.

### 4.2. Model Verification in MSC Adams

All the simulations up to this point were performed using the model we developed (described in Section 3). This model has disadvantages and limitations due to its basic properties and its assumptions. Before we continue to more advanced simulations and experiments, we validate our model by reconducting some of the simulations using the commercial multibody dynamic simulator MSC Adams™ (Version 2019).

**Figure 5:**
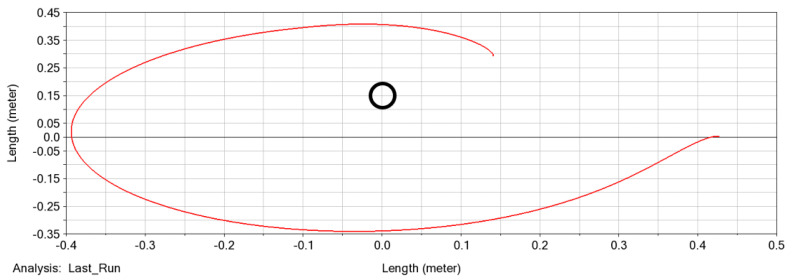
Simulation with fixed obstacles in MSC Adams™. The red line represents the end-effector trajectory, and the black circle represents the obstacle. The simulation duration is 0.5 s. The trajectory is similar to the one we achieved with our own framework.

The first simulation was designed to validate our contact model in the presence of obstacles in the environment. For this purpose, an obstacle with a radius of 5 cm was positioned at $\left(0,0.2\right)\;m$. An input torque of 0.085 N·m was applied to the base of the manipulator. The results can be seen in Figure 5, and it is noteworthy that the end-effector changes its axis of rotation upon contacting the obstacle. These results are comparable to the results presented in Figure 1, which were obtained using our original simulation framework.

**Figure 6:**
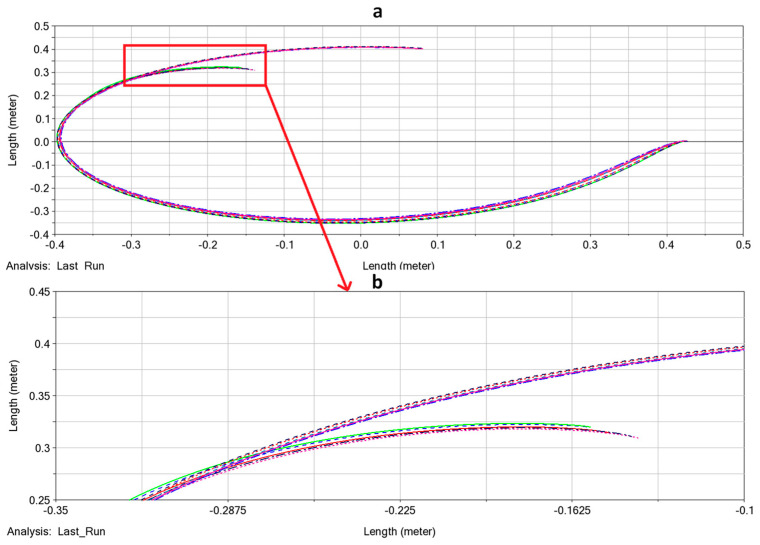
Effect of initial condition uncertainty on the system’s robustness during obstacle interaction in MSC Adams™. (**a**) Trajectories in Cartesian plane under initial condition uncertainty (i.e., initial torque and angle). The lines represent the end-effector trajectory. (**b**) Zoom in on the trajectories at the moment of interaction with the obstacle.

The next simulation was conducted to validate the behavior of the system under uncertainty in the initial conditions. For this simulation, we use Figure 2 as a reference simulation that was made with the model described in Section 3. To this end, a set of ten simulations were performed: five simulations with an obstacle positioned at $\left(0.141,0.141\right)\;m$ and five simulations without obstacles. The results are depicted in Figure 6. Although the results in Adams are slightly less clear, it is still evident that the uncertainty in the end-effector position is reduced due to the interaction with the obstacle.

The aforementioned simulations were conducted to validate our mathematical model and simulation framework. The strong correlation between the results obtained from the two different environments gives us confidence that our model is accurate and functioning as expected.

### 4.3. Movable Obstacles

In all simulations performed until now, the obstacles within the environment were fixed, and the input to the system consisted of the torque command and initial angle of the manipulator. In this section, we aim to set the torque command and initial angle as constants in all simulations, while the genetic algorithm generates the position and radius of obstacles. Each simulation is provided with a distinct target configuration comprising the end-effector position, velocity vector, and orientation vector.

**Figure 7:**
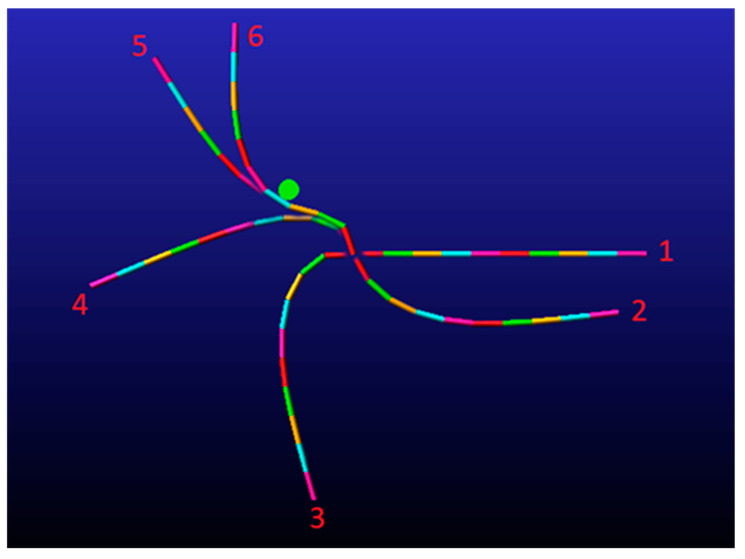
Simulation of a multilink underactuated manipulator interacting with a movable obstacle (shown as green circle). The manipulator sequence over time is denoted by the numbers at the ends of the manipulator. The simulation duration is 0.5 s. The end-effector does indeed pass through its target point in the desired velocity and orientation vector.

All simulations in this section were performed using MSC Adams™ and not with the original simulation. In the first simulation, the end-effector was assigned to traverse through the point $\left(-0.18,0.35\right)$ with a velocity vector of $\left(1,0\right)$ and an orientation vector of $\left(0,1\right)$. The velocity vector only represents the velocity direction, and its magnitude is not significant in the cost function as long as the direction is correct. In this scenario, the velocity vector denotes a positive velocity in the *x*-axis, while the orientation vector represents the normal vector that determines the end-effector angle. In this instance, the end-effector was directed to align with the *y*-axis. The output of the algorithm was to place an obstacle with a radius of 1.6 cm at the coordinates $\left(-0.1,0.1\right)$. Figure 7 displays the outcome of the simulation, indicating that the end-effector indeed traversed through the designated point while moving from left to right (i.e., along the positive *x*-axis) and aligning with the positive *y*-axis.

**Figure 8:**
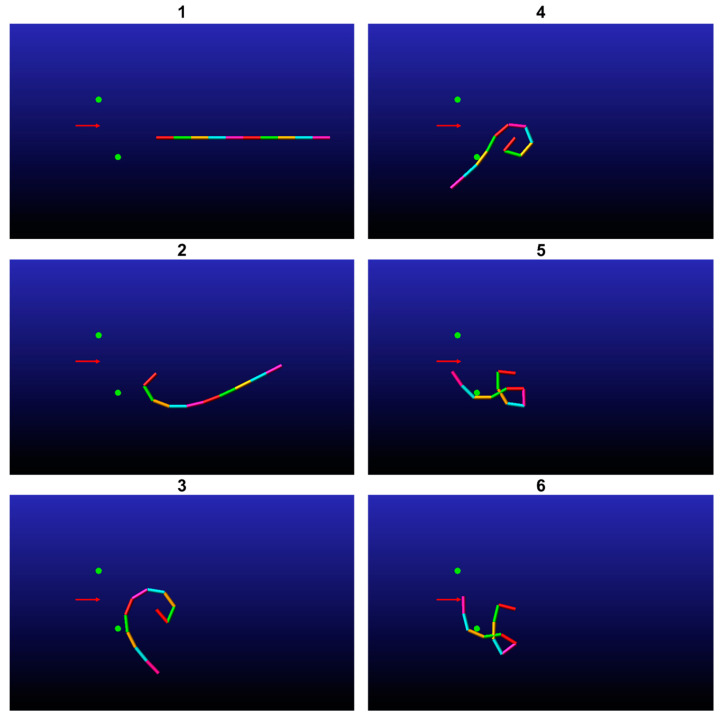
A simulation of a multilink underactuated manipulator interacting with movable obstacles (marked by green circles). The manipulator sequence over time is denoted by the number at the top of each figure. The end-effector does indeed pass through its target point in the desired velocity and orientation vector.

In the second simulation, the end-effector was programmed to traverse through the point $\left(-0.2,0\right)$ with a velocity vector of $\left(1,0\right)$ and an orientation vector of $\left(1,0\right)$. To enable the manipulator to accomplish its task, the damping coefficient was reduced. This simulation includes two obstacles. The outcome of the algorithm was to place the first obstacle at $\left(-0.1,-0.05\right)$ and the second at $\left(-0.1,0.1\right)$, with both obstacles having a radius of 0.8 cm. Figure 8 illustrates six snapshots of the simulation, which demonstrate that the end-effector was able to complete its task. It is worth noting that the manipulator did not interact with one of the obstacles as part of the solution.

**Figure 9:**
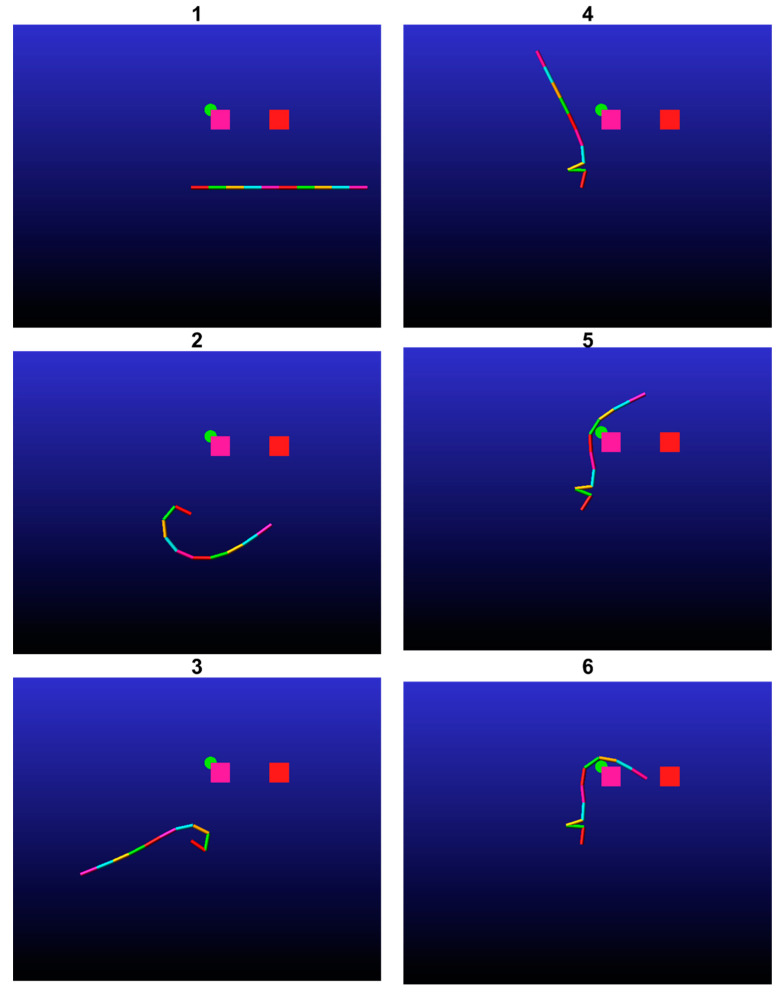
A multilink underactuated manipulator interacting with a movable obstacle (marked by green circle) and passing between two target points. The manipulator sequence over time is denoted by the number at the top of each figure. The end-effector does indeed pass through its target point.

In the third simulation, the objective of the genetic algorithm was to identify a solution that would enable the end-effector to traverse between two adjacent obstacles without touching them while the end-effector velocity was along the negative *y*-axis. The algorithm’s output was to position an obstacle with a radius of 1.6 cm at $\left(0.05,0.2\right)$. Figure 9 presents six snapshots of the simulation result.

These three simulations indicate that the manipulator can accomplish more tasks and attain target positions and orientations that were previously unattainable by regulating the environment and obstacle positions.

## 5. Experiments

### 5.1. Experimental Setup

**Figure 10:**
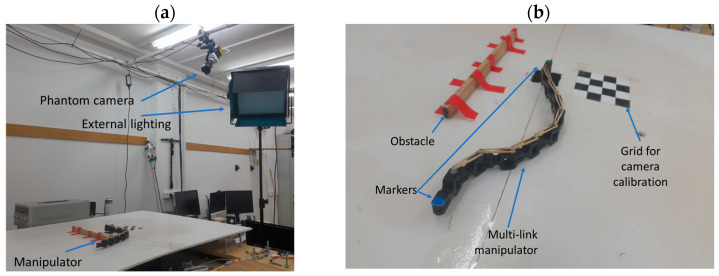
The experimental setup for studying manipulation using a multilink manipulator. (**a**) Wide view of the experimental setup showing the manipulator, camera, and external lighting. (**b**) Close-up view of the deformable manipulator and the obstacle, along with the calibration pattern.

To validate the theoretical results of our simulations, we conducted real hardware experiments using an experimental setup comprising several key subsystems: a multilink manipulator, an actuation system, and a data-gathering system. In this chapter, we review each of these subsystems in detail. Figure 10 shows an overview of the experimental setup, with the upper part (Figure 10a) displaying a wide-angle view that includes the multilink manipulator, the motion-capture system, and external lighting. A Supplementary Video showcases some of the results and additional information.

The multilink manipulator used in the experiments is comprised of 10 3D-printed links made of PLA, with four ball bearings incorporated into each link to minimize the friction in the joints and maintain precise mechanical tolerances.

As previously mentioned, the multilink manipulator is actuated by a single rotational motor positioned at the base of the manipulator. We employed a DC brushed motor with a gear ratio of 1:80 to achieve a high torque at low RPM. We used a motion driver (Elmo SOLO-Twitter G-SOLTWI10/100EE7) to control the motor. Since the motor current is proportional to the applied torque, we utilized the current loop to closely match the experimental system to the theoretical model. Our motor system operates on a 24 V power supply and can consume up to 5 A of current. The driver is controlled through an RS-232 interface from a computer, which runs a MATLAB program (version 2020b) to manage the entire process. The control loop gains were calibrated using Elmo software (https://elmosoftware.com.au/). The sensing system comprises a Phantom™ Miro LAB-320 high-speed camera with a 20 mm F1.4 SIGMA™ lens, which records at 120 Hz and a 1024 × 768 resolution. The camera is used to analyze the system’s behavior offline. In addition, a checkerboard is used to calibrate skews in the image, and different markers in blue, red, and green are employed to identify the locations of the end-effector, obstacle, and motor origin, respectively. Figure 10b shows the multilink manipulator and obstacle, along with the markers and the checkerboard.

### 5.2. Experiments with Fixed Obstacles

Our first objective was to examine the behavior of the manipulator during its interaction with a fixed obstacle while accounting for uncertainty in the initial conditions of the system, as illustrated in Figure 2. To this end, we conducted a set of 25 experiments, comprising 20 trials with a single, fixed obstacle present in the environment and 5 trials with no obstacles at all. We introduced variations in the initial angle and torque command and added noise of up to 30% in these parameters.

**Figure 11:**
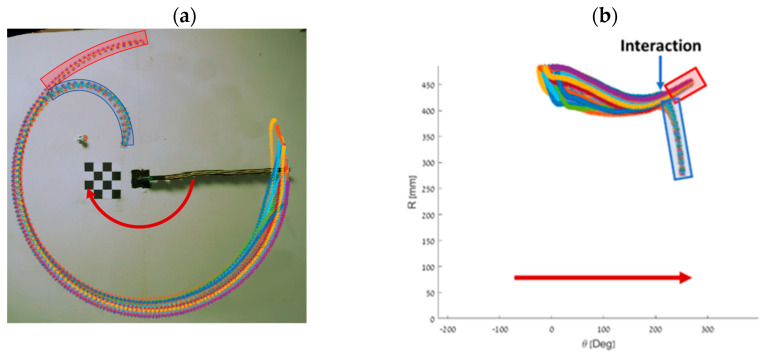
Comparison of trajectories with and without obstacle interaction. (**a**) Trajectories from 24 experiments, including 19 with a single obstacle (blue box) and 5 without any obstacle (red box). Overlaid snapshots of the manipulator are shown above. (**b**) Polar representation of the trajectories from the 24 experiments. The blue box shows trajectories that interacted with the obstacle, while the red box shows trajectories that did not interact with the obstacle.

In Figure 11a, the red arrow indicates the motion direction, while the small red circle indicates the obstacle’s location. As expected, due to the interaction with the obstacle, the motion radius decreases. Upon examining the slow-motion recording, we verified that the relative velocity between the obstacle and the contact point is indeed zero, confirming the reliability of our contact model. Figure 11b presents the same data set using a polar coordinate system, where the *x*-axis shows the angle from the origin to the end-effector with respect to the world frame *x*-axis, and the vertical represents the radial distance from the origin to the end-effector. The blue and red boxes correspond to the interacting and noninteracting trajectories, respectively. We observe that the variance between the trajectories significantly reduces after the interaction with the obstacle. Another important result from this experiment is the verification of the dynamic model and the plastic impact assumption.

**Figure 12:**
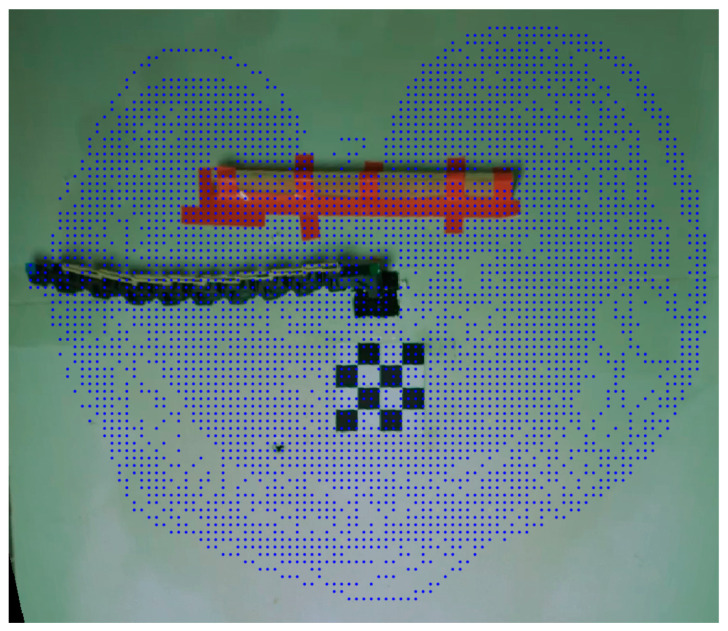
The experimental workspace of the manipulator superimposed on a snapshot of the environment. The manipulator’s reach is shown in blue. A single obstacle is present in the environment.

The goal of the next experiment was to estimate the workspace of a multilink manipulator. A single obstacle with a length of 0.4 m was randomly placed in the environment, and the manipulator was subject to a random motion. We gathered over 100,000 data points, which we later organized into a grid for convenience. The results are presented in Figure 12. We can observe an additional area behind the obstacle that the manipulator was able to reach, which a rigid manipulator would have been unable to access.

**Figure 13:**
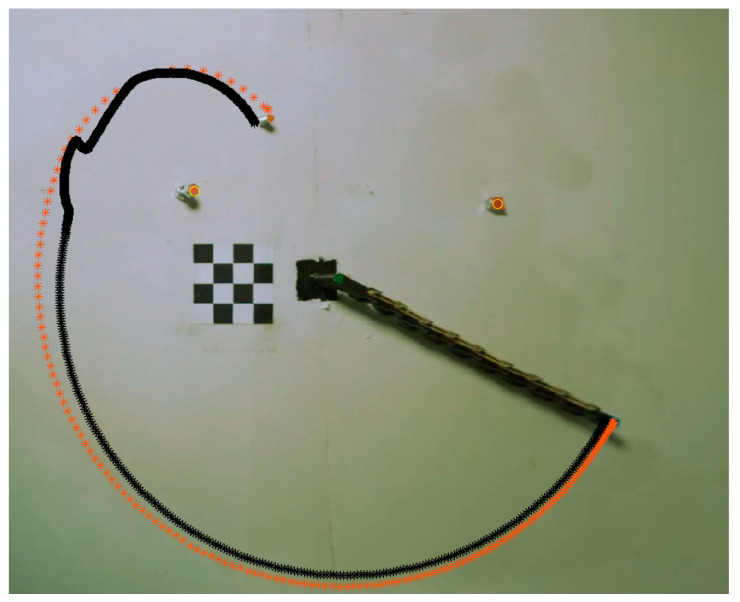
Comparison of the experimental (in orange) and simulated (in black) trajectories of the end-effector. The trajectories depict the end-effector passing through the intended target point.

In the final experiment, we demonstrate a complete autonomous pick-up process, depicted in Figure 13. We randomly selected an environment configuration that includes two fixed obstacles. First, a camera captures a snapshot of the environment, followed by a simple computer vision algorithm that recognizes the obstacles and target location by their color markers. This allows for the correct positions to be updated in the simulation environment. Then, the genetic algorithm is used to find a fitting torque command to reach the target, using the predetermined initial angle extracted from the initial scan. The parameters for the genetic algorithm are the same as in Section 4. After 50 iterations, the algorithm converged and sent the motion command for the motor through the driver, while the camera captured the trajectory of the end-effector. The results of the trajectory from the simulation and experiment are presented in Figure 8. As anticipated, the end-effector passed through the target point and collected the target.

### 5.3. Experiments with Movable Obstacles

**Figure 14:**
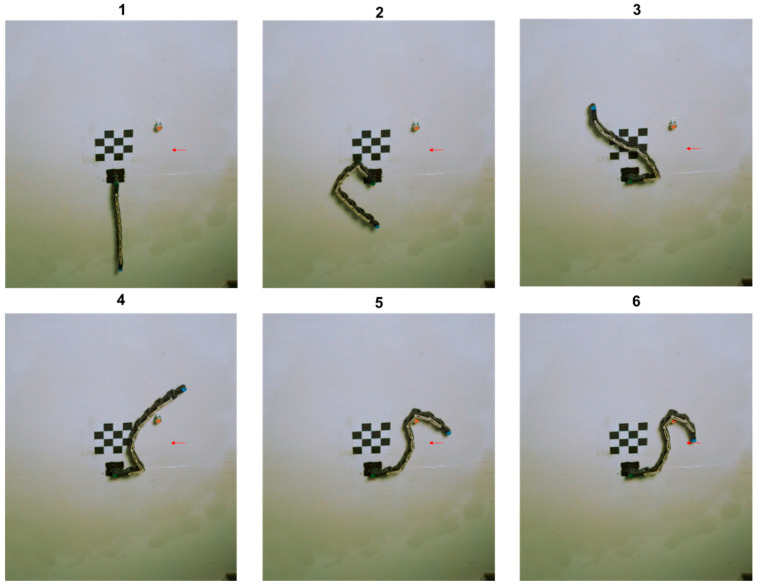
Experiment with a movable obstacle. In this experiment, the desired position and velocity vector were set, and a genetic algorithm returns a solution of the obstacle’s position and radius to achieve this manipulator configuration. As can be seen, the end-effector indeed passes through the target point in the desired velocity vector.

In the subsequent phase, we validate, through an experiment, the simulation outcomes of manipulation in movable environments as presented earlier. To this end, the same experimental arrangement as in the previous section was utilized. In the first experiment, we demonstrate the end-effector passing through a point with a velocity along the negative *y*-axis. It is evident that such motion at that particular point is unattainable without the use of obstacles. Hence, we can infer that by utilizing movable obstacles, we can amplify the feasible orientations for a given point in the workspace. The experiment results are presented in Figure 14.

This method for this experiment is similar to the one presented in the related simulations section. The target position and velocity vector are defined as inputs for the genetic algorithm, and by controlling the obstacle position and radius, the algorithm minimizes the cost function, which is defined as

(7) $$CostFunction=min\left(\left\|P_{i}-T_{i}\right\|\right)\;\forall i\in S\left(0,1\right).$$

As presented in Figure 14, the algorithm indeed manages to converge to the desired solution and brings the end-effector to the desired configuration.

## 6. Conclusions and Future Work

This paper presented a study on the manipulation of bio-inspired multilink manipulators in environments containing obstacles. Our bio-inspired manipulator not only used a low number of actuators, but also worked in environments with obstacles. A theoretical model was developed for the multilink manipulator that included its interaction with obstacles. This model was tested through various simulations that assessed the system’s robustness to uncertainty in both the initial conditions and the obstacle positions. The experimental setup was then described, and the experiments conducted were reviewed. Notably, the work included a complete process of picking up a target object as a demonstration of the system’s capabilities.

The experiments we conducted are consistent with the theoretical model we developed. Despite using a minimalistic approach with only a single active actuator, the system’s performance suggests that this type of setup is worth considering for certain applications, particularly in environments with obstacles.

In this work, it was demonstrated how the uncertainty in the end-effector’s position converges in 0.1 s, and the standard deviation decreases to 28% of the initial value, measured before the interaction. The robustness of the system was examined by applying noise to the initial conditions with a magnitude of up to 50%, while still converging to the desired target position. Next, an effective method for trajectory planning was introduced and was tested in various scenarios including target orientation, in addition to the target position. The complete workspace of the manipulator was tested as well and was found to be similar to the simulated one.

Our goal is to further our understanding of the system’s behavior by performing a parametric investigation, with the aim of studying the impacts of different parameters such as rope damping and spring coefficients. We also plan to expand this work to include elastic materials, which can undergo finite length changes under external forces. By utilizing elastic materials in environments with and without obstacles, we expect to be able to exploit an even larger workspace and achieve a significant advantage over traditional rigid manipulators.

## Figures and Tables

**Table 1:** Summary of the impact of different noise amplitudes on the variance of trajectories and their convergence rates. The table presents the maximum standard deviation (SD) along the trajectory parameterization S and the difference in the SD before and after interaction with the obstacle.

Noise Amplitude	SD Max	SD Difference
10%	0.01	0.005
25%	0.015	0.003
33%	0.018	0.004
50%	0.02	0.005

## Data Availability

The original contributions presented in the study are included in the article/Supplementary Material; further inquiries can be directed to the corresponding author/s.

## Supplementary Materials

The following supporting information can be downloaded at: https://www.mdpi.com/article/10.3390/biomimetics9020086/s1, Video S1: Short video demonstrating the manipulator in action.

## References

[1] Hooper S.L. (2021). Motor control: Elephant trunks ignore the many and choose a few. Curr. Biol..

[2] Dagenais P., Hensman S., Haechler V., Milinkovitch M.C. (2021). Elephants evolved strategies reducing the biomechanical complexity of their trunk. Curr. Biol..

[3] Prigozin A., Degani A. A Minimalistic Hyper-Flexible Manipulator: Modeling and Control. Proceedings of the 2020 IEEE/RSJ International Conference on Intelligent Robots and Systems (IROS).

[4] Wilson J.F., Mahajan U., Wainwright S.A., Croner L.J. (1991). A continuum model of elephant trunks. J. Biomech. Eng..

[5] Mishra M.K., Samantaray A.K., Chakraborty G., Jain A., Pathak P.M., Merzouki R. Dynamic modelling of an elephant trunk like flexible bionic manipulator. Proceedings of the ASME 2019 International Mechanical Engineering Congress and Exposition.

[6] Kang M., Han Y.-J., Han M.-W. (2023). A Shape Memory Alloy-Based Soft Actuator Mimicking an Elephant’s Trunk. Polymers.

[7] Huang Q., Wang P., Wang Y., Xia X., Li S. (2022). Kinematic Analysis of Bionic Elephant Trunk Robot Based on Flexible Series-Parallel Structure. Biomimetics.

[8] Nguyen V.P., Dhyan S.B., Mai V., Han B.S., Chow W.T. (2023). Bioinspiration and Biomimetic Art in Robotic Grippers. Micromachines.

[9] Lin C., Chang P., Luh J. (1983). Formulation and Optimization of Cubic Polynomial Joint Trajectories for Industrial Robots. IEEE Trans. Autom. Control..

[10] Kyriakopoulos K.J., Saridis G.N. Minimum Jerk Path Generation. Proceedings of the 1988 IEEE International Conference on Robotics and Automation.

[11] Zavlangas P.G., Tzafestas S.G. (2000). Industrial robot navigation and obstacle avoidance employing fuzzy logic. J. Intell. Robot. Syst..

[12] Rubio F., Llopis-Albert C., Valero F., Suñer J.L. (2016). Industrial robot efficient trajectory generation without collision through the evolution of the optimal trajectory. Robot. Auton. Syst..

[13] Suzuki T., Ebihara Y., Ando Y., Mizukawa M. Casting and winding manipulation with hyper-flexible manipulator. Proceedings of the 2006 IEEE/RSJ International Conference on Intelligent Robots and Systems.

[14] Yamakawa Y., Namiki A., Ishikawa M. Dexterous manipulation of a rhythmic gymnastics ribbon with constant, high-speed motion of a high-speed manipulator. Proceedings of the 2013 IEEE International Conference on Robotics and Automation (ICRA).

[15] Yamakawa Y., Namiki A., Ishikawa M. Motion planning for dynamic folding of a cloth with two high-speed robot hands and two high-speed sliders. Proceedings of the IEEE International Conference on Robotics and Automation.

[16] Yamakawa Y., Namiki A., Ishikawa M. Motion planning for dynamic knotting of a flexible rope with a high-speed robot arm. Proceedings of the 2010 IEEE/RSJ International Conference on Intelligent Robots and Systems.

[17] Mochiyama H., Nakajima H., Hatakeyama T. Chameleon-like shooting manipulator for accurate 10-meter reaching. Proceedings of the 2015 10th Asian Control Conference (ASCC).

[18] Fagiolini A., Belo F.A.W., Catalano M.G., Bonomo F., Alicino S., Bicchi A. Design and control of a novel 3D casting manipulator. Proceedings of the 2010 IEEE International Conference on Robotics and Automation.

[19] Hill L., Woodward T., Arisumi H., Hatton R.L. Wrapping a target with a tethered projectile. Proceedings of the 2015 IEEE International Conference on Robotics and Automation (ICRA).

[20] Ito K., Yamakawa Y., Ishikawa M. Winding manipulator based on high-speed visual feedback control. Proceedings of the 2017 IEEE Conference on Control Technology and Applications (CCTA).

[21] Li X., Yue H., Yang D., Sun K., Liu H. (2023). A Large-Scale Inflatable Robotic Arm Toward Inspecting Sensitive Environments: Design and Performance Evaluation. IEEE Trans. Ind. Electron..

[22] Li X., Sun K., Guo C., Liu H. (2022). Hybrid adaptive disturbance rejection control for inflatable robotic arms. ISA Trans..

[23] Shapiro Y., Wolf A., Gabor K. (2011). Bi-bellows: Pneumatic bending actuator. Sens. Actuators A Phys..

[24] Hertz H. (1881). Über die berührung fester elastischer körper. J. Für Die Reine Und Angew. Math..

[25] Goodman L.E. (1960). Contact stress analysis of normally loaded rough spheres. J. Appl. Mech. Trans. ASME.

[26] Mossakovskii V. (1963). Compression of elastic bodies under conditions of adhesion (Axisymmetric case). J. Appl. Math. Mech..

[27] Spence D.A. (1968). Self similar solutions to adhesive contact problems with incremental loading. Proc. R. Soc. London. Ser. A Math. Phys. Sci..

[28] Spence D.A. (1975). The hertz contact problem with finite friction. J. Elast..

[29] Greenwood J.A., Williamson J.B.P. (1966). Contact of nominally flat surfaces. Proc. R. Soc. London. Ser. A Math. Phys. Sci..

[30] Goldberg D.E., Lingle R. Alleles, loci, and the traveling salesman problem. Proceedings of the First International Conference on Genetic Algorithms and Their Applications.

